# Supplementary material for: The ctenophore Mnemiopsis leidyi deploys a rapid injury response dating back to the last common animal ancestor
Source: Commun Biol. 2024 Feb 19;7:203. doi: 10.1038/s42003-024-05901-7 (PMC10876535; doi:10.1038/s42003-024-05901-7)
Supplement: Supplementary file 2 — Supplementary Information [file 42003_2024_5901_MOESM2_ESM.pdf]

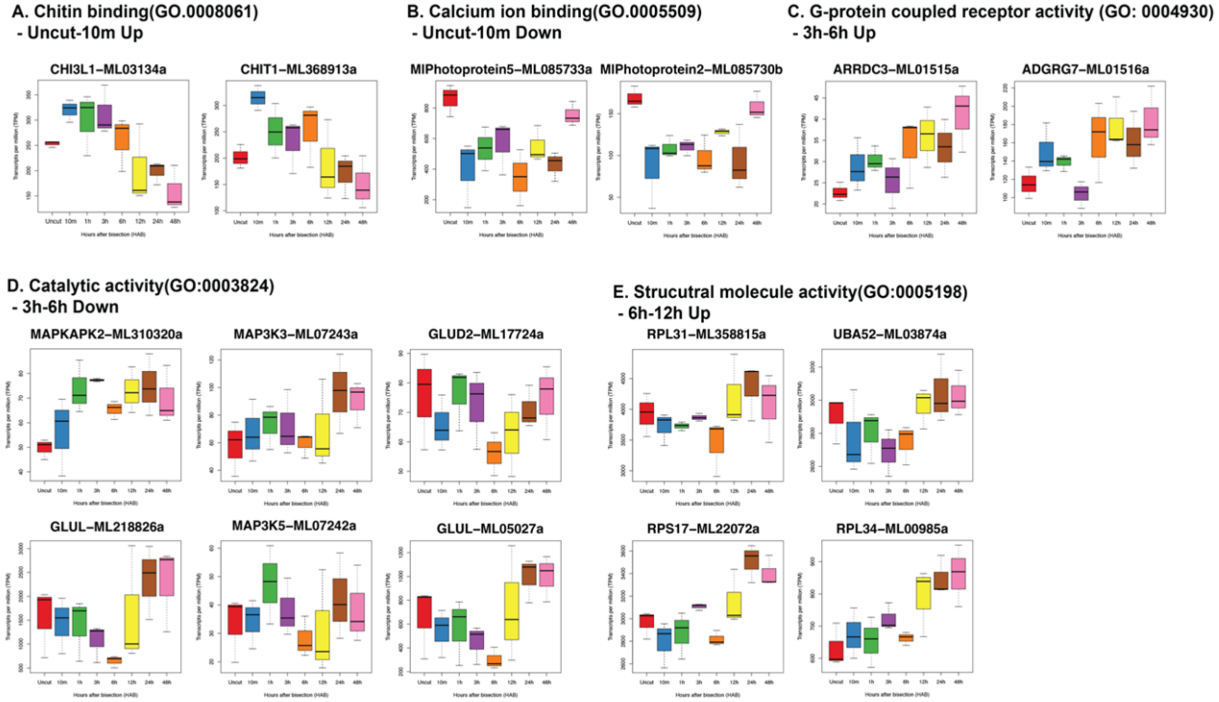

Supplementary Figure 1. Expression of individual DEG extracted from enriched GO terms in specified intervals across the time course. X axes include time points from Uncut-48 hours post bisection(hpb). Y axes show expression level in transcripts per million(TPM).

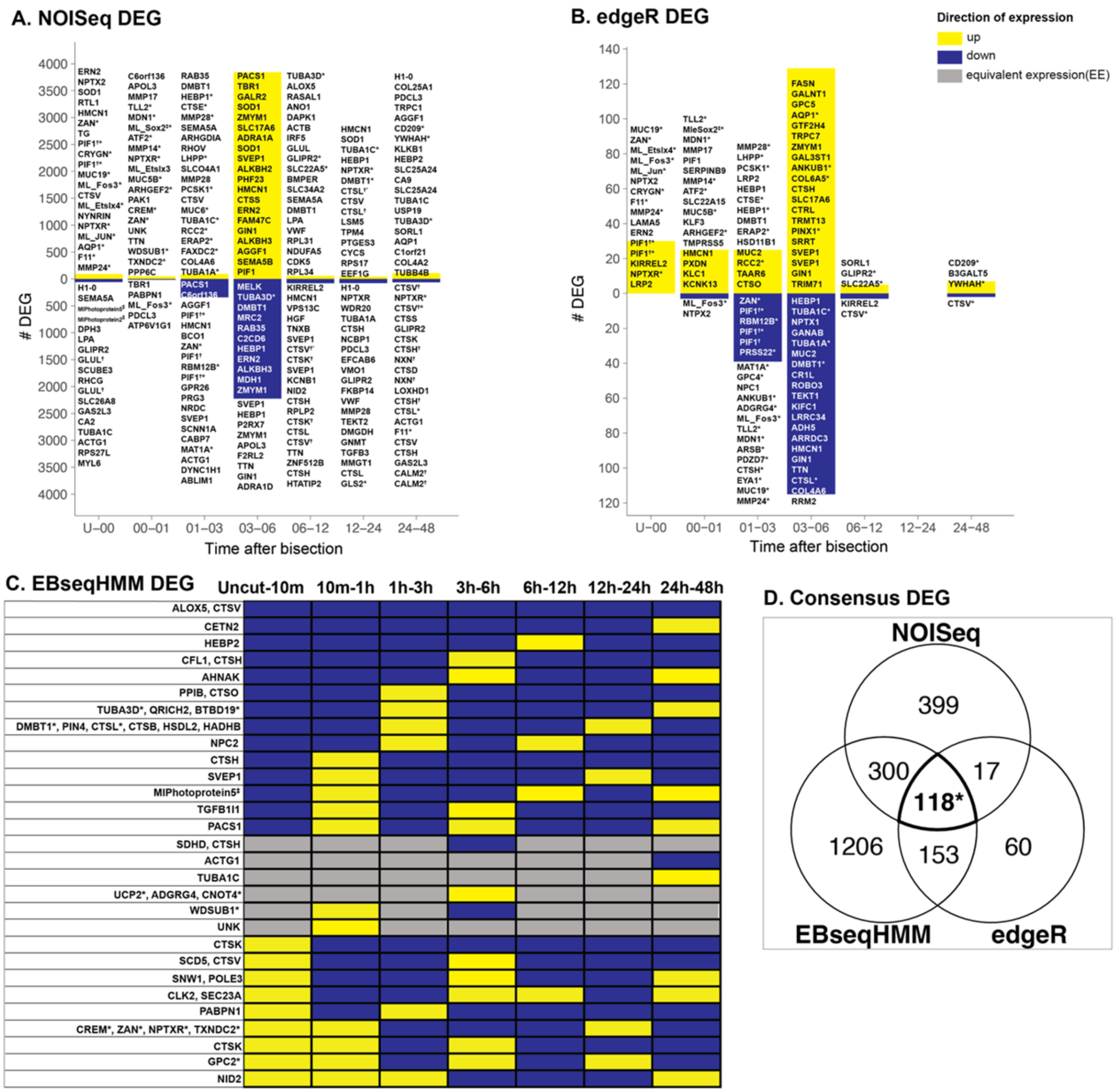

Supplementary Figure 2. A-B. Summary of DEG in pairwise methods (NOISeq and edgeR). Bar graphs showing the total quantity of DEG from NOISeq (A.) and edgeR (B.) on the y-axis separated into upregulated (yellow, above x axis) and downregulated (blue, below x axis) in each successive time interval of NOISeq. Lists of BLAST annotations of DEG are overlaid on bars ordered by A. absolute log2fold change from greatest to least and B. p-value from least to greatest. Gene nomenclature is based on best BLAST hit to the human protein database unless we performed phylogeny. C. DEG identified by EBseq-hmm with maximum posterior probability (PP) greater than 0.5 were classified as high-confidence assignments, reducing the result to 102 genes and 43 expression patterns. Protein annotations were assigned by BLAST to the human database for 48 out of the 102 DEG (posterior probability > 0.5), which were categorized into 29 expression paths. Uncut, 10m = 10 minutes post bisection (mpb), 1h-48h = hours post bisection (hpb). Gene nomenclature is based on best BLAST hit to the

human protein database unless we performed phylogeny (Supplementary File 2). †Multiple *M. leidyi* genes received identical best BLAST hit. ‡ ML_Sox2 (ML234028a) annotation derived from Schnitzler et al (2014). MlPhotoprotein5 (ML085733a) and MlPhotoprotien2 (ML085730b) annotation derived from Schnitzler et al (2012). *Genes (according to their corresponding *M. leidyi* gene ID) are included in the consensus between the three methods. D. Venn diagram showing the number of genes found between the three methods.

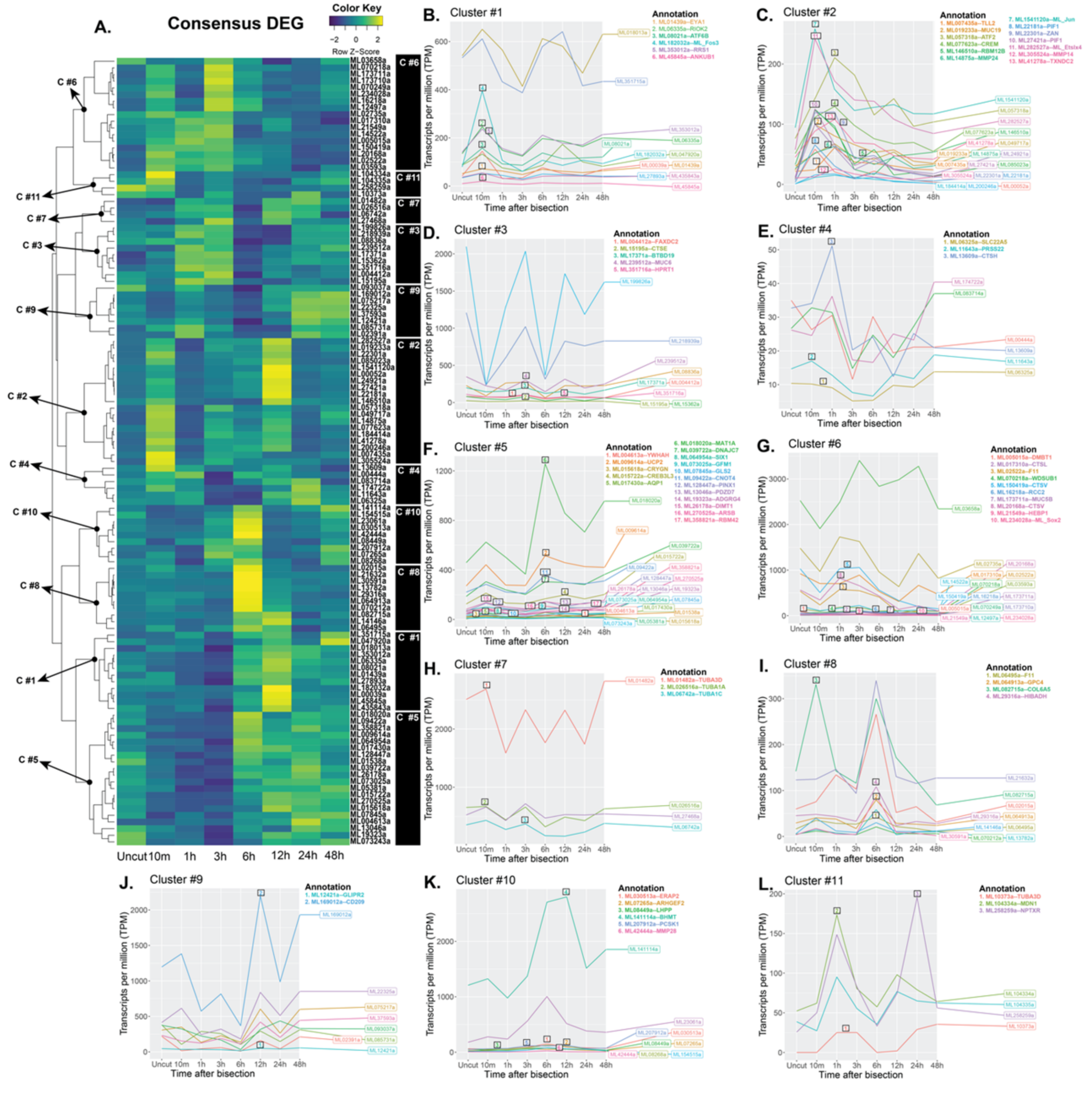

Supplementary Figure 3. Heatmap and cluster visualization of consensus DEG. A. Heatmap of 118 consensus DEG with sequential time points on x axis and *M. leidyi* gene IDs ordered according to cluster membership on y axis. Boxes are colored according to respective row z-score.

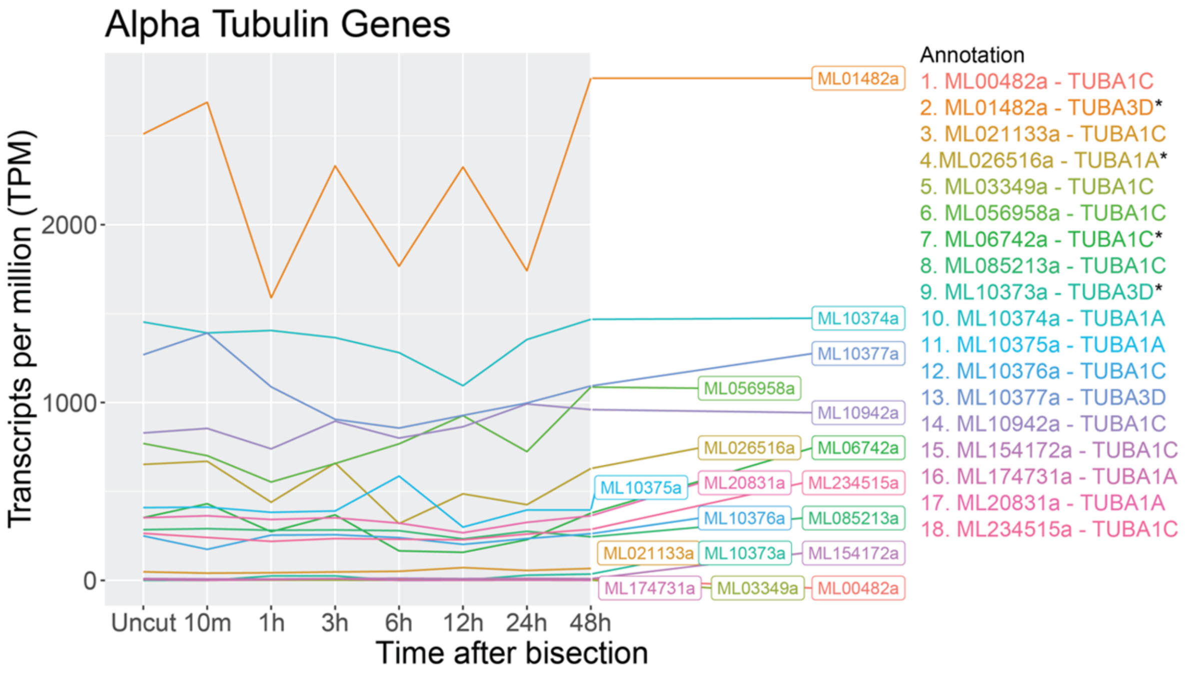

Supplementary Figure 4. The 18 alpha tubulin genes found in the M. leidyi protein models BLAST reference (Supplementary File 2). The median expression across biological replicates is mapped across each timepoint. *Genes are included in the consensus between the three methods.

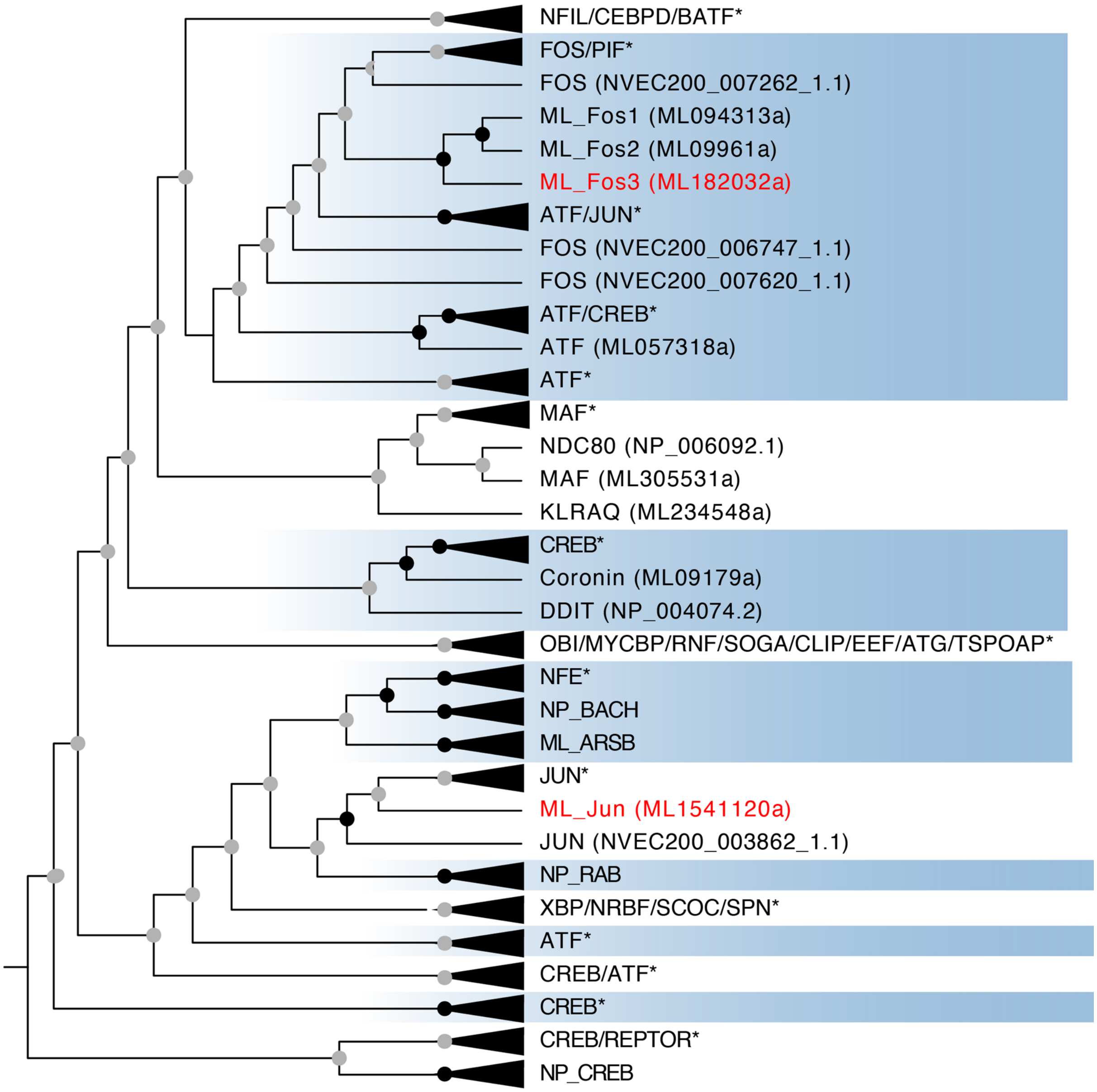

Supplementary Figure 5. Maximum likelihood tree of bZIP domain-containing genes across *Nematostella*, *Drosophila,* humans and *Mnemiopsis*. Black circles indicate a node with a bootstrap value greater than 95. ML = *Mnemiopsis*, NP = human, FB = *Drosophila* (nonvisible in this collapsed visualization), NVEC = *Nematostella*. *Grouping includes multiple species. Blue gradient delineates major families.

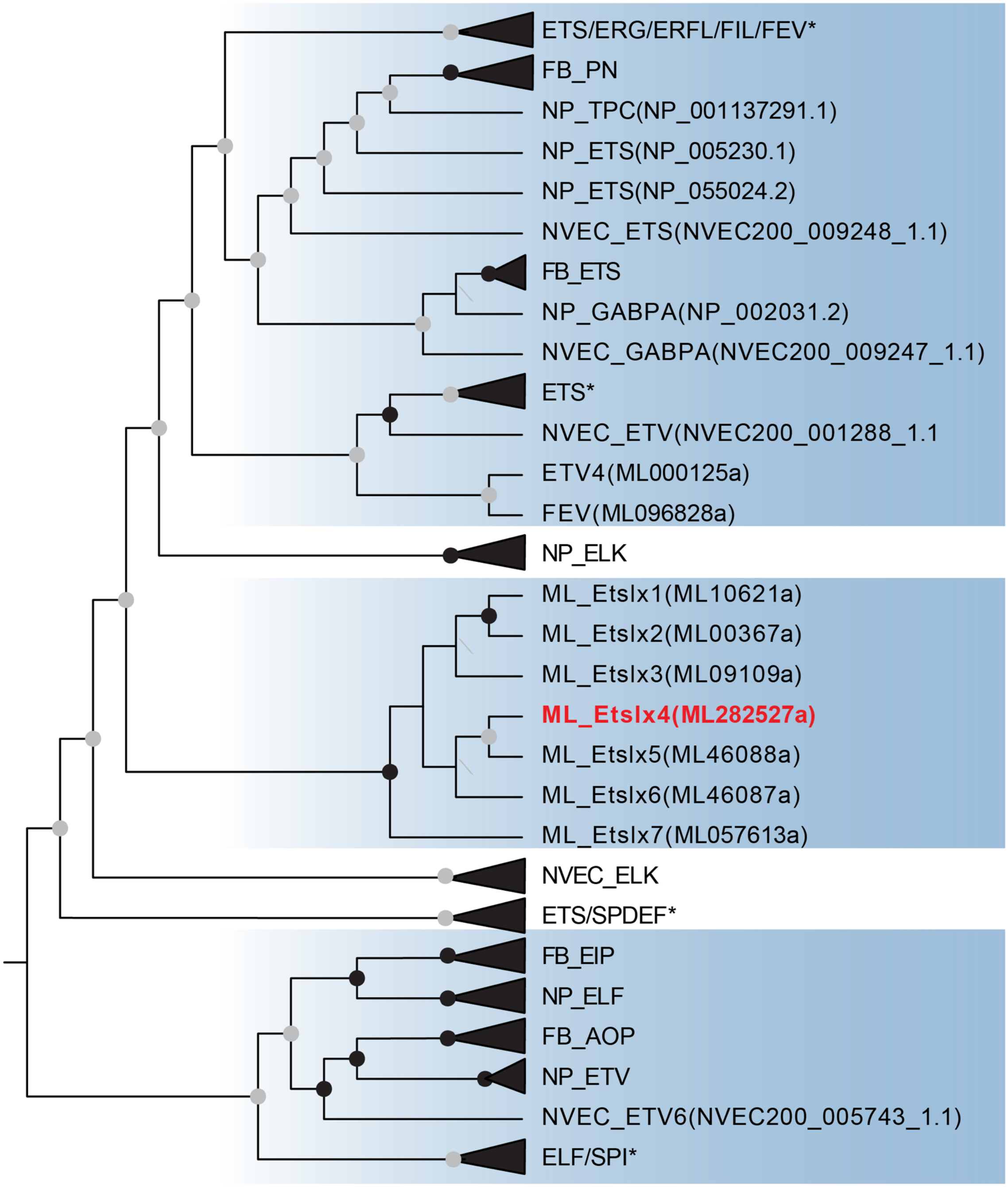

Supplementary Figure 6. Maximum likelihood tree of ETS domain-containing genes across *Nematostella*, *Drosophila,* humans and *Mnemiopsis*. Black circles indicate a node with a bootstrap value greater than 95. ML = *Mnemiopsis*, NP = human, FB = *Drosophila*, NVEC = *Nematostella*. *Grouping includes multiple species, see Supplementary file X for full gene list. Blue gradient delineates major families.

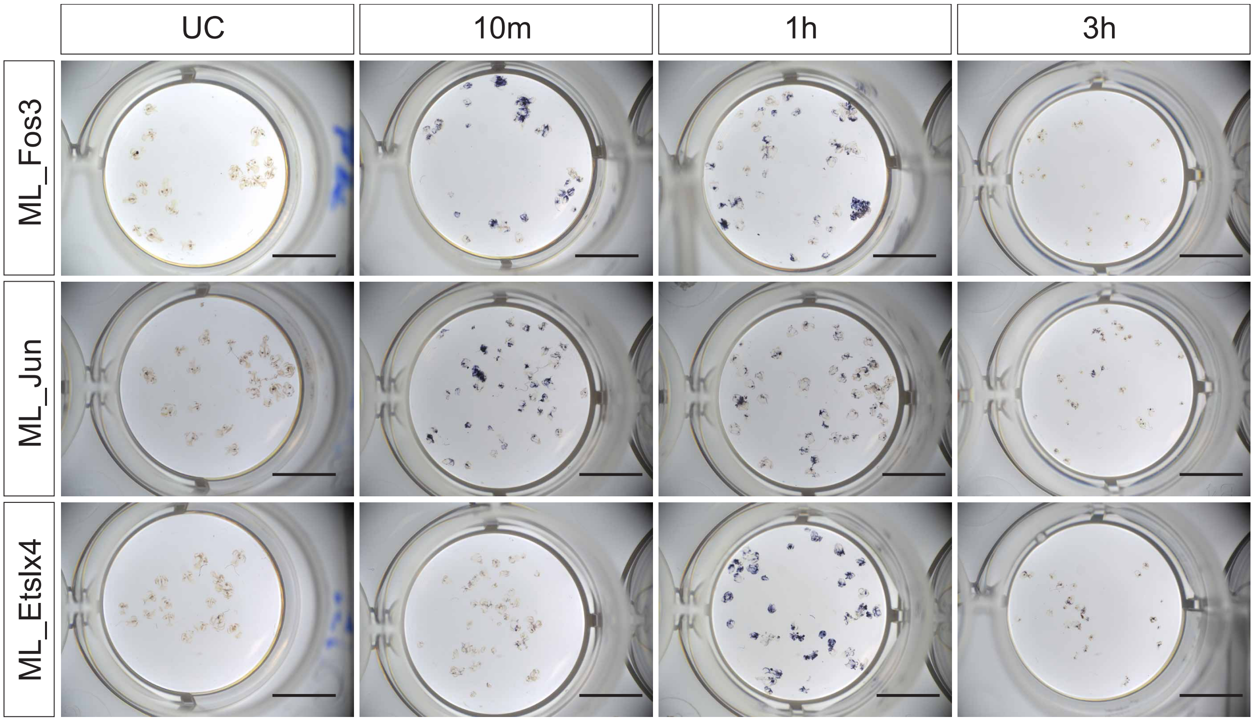

Supplementary Figure 7. Wells from insitu hybridization experiments using anti-sense probes. Gene nomenclature is based on phylogeny. UC = Uncut ,10m = 10 minutes post bisection (mpb), 1h-48h= hours post bisection (hpb).

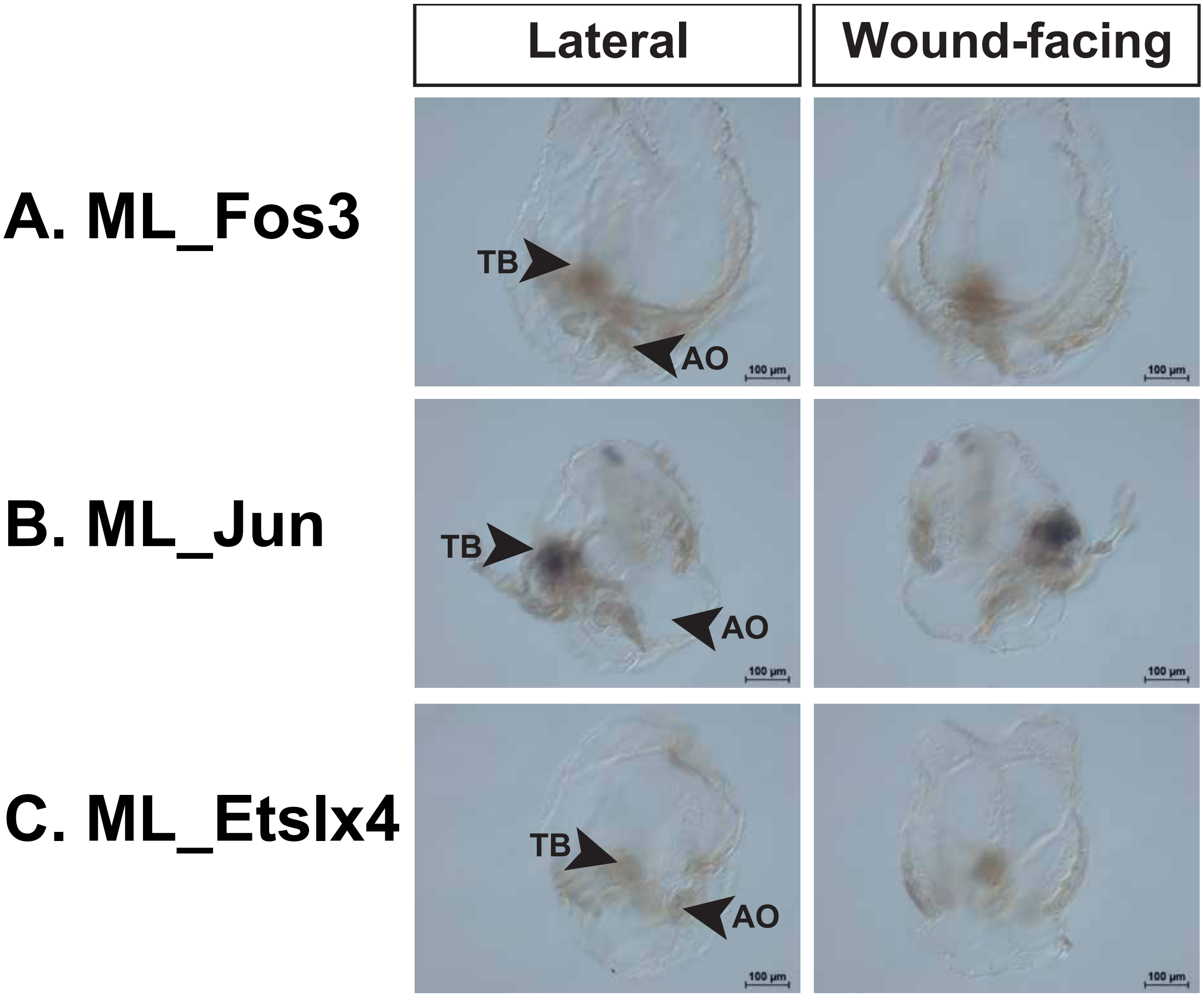

Supplementary Figure 8. High magnification of sense-transcribed control probes. Gene nomenclature is based on phylogeny. All are 10mpb.
