# Supplementary material for: The ctenophore Mnemiopsis leidyi deploys a rapid injury response dating back to the last common animal ancestor
Source: Commun Biol. 2024 Feb 19;7:203. doi: 10.1038/s42003-024-05901-7 (PMC10876535; doi:10.1038/s42003-024-05901-7)
Supplement: Supplementary file 3 — Description of Additional Supplementary Files [file 42003_2024_5901_MOESM3_ESM.pdf]

Description of Additional Supplementary Files

File name: Supplementary Data 1
Description: FastQC reports of raw and trimmed reads.

File name: Supplementary Data 2
Description: Quantification of genes from RSEM.

File name: Supplementary Data 3
Description: Reciprocal best BLAST hit for each M. leidyi gene model and corresponding e-value.

File name: Supplementary Data 4
Description: Lists of DEG from each method, used to generate the consensus DEG.

File name: Supplementary Data 5
Description: DEG Lists with p-vales/log2FC values from EdgeR, NOISeq, and EBSeqHmm as shown in Supplementary Figure 2.

File name: Supplementary Data 6
Description: Enriched terms for each time interval and each direction (Up or Down). Under each interval contains the ML gene associated with that term and its corresponding reciprocal best BLAST hit.

File name: Supplementary Data 7
Description: Tree files associated with the BZIP and ETS protein family trees (see Supplementary Figure 5 and 6).

File name: Supplementary Data 8
Description: RNA probe sequences aligned to ML gene models.
