# Supplementary material for: The ctenophore Mnemiopsis leidyi deploys a rapid injury response dating back to the last common animal ancestor
Source: Commun Biol. 2024 Feb 19;7:203. doi: 10.1038/s42003-024-05901-7 (PMC10876535; doi:10.1038/s42003-024-05901-7)
Supplement: Supplementary file 4 — Reporting Summary [file 42003_2024_5901_MOESM4_ESM.pdf]

nature portfolio

Corresponding author(s): Mark Q. Martindale

Last updated by author(s): Jan 23, 2024

Reporting Summary

Nature Portfolio wishes to improve the reproducibility of the work that we publish. This form provides structure for consistency and transparency in reporting. For further information on Nature Portfolio policies, see our Editorial Policies and the Editorial Policy Checklist.

Statistics

For all statistical analyses, confirm that the following items are present in the figure legend, table legend, main text, or Methods section.

n/a	Confirmed	
☐	☒	The exact sample size (*n*) for each experimental group/condition, given as a discrete number and unit of measurement
☐	☒	A statement on whether measurements were taken from distinct samples or whether the same sample was measured repeatedly
☐	☒	The statistical test(s) used AND whether they are one- or two-sided<br>*Only common tests should be described solely by name; describe more complex techniques in the Methods section.*
☐	☒	A description of all covariates tested
☐	☒	A description of any assumptions or corrections, such as tests of normality and adjustment for multiple comparisons
☐	☒	A full description of the statistical parameters including central tendency (e.g. means) or other basic estimates (e.g. regression coefficient) AND variation (e.g. standard deviation) or associated estimates of uncertainty (e.g. confidence intervals)
☐	☒	For null hypothesis testing, the test statistic (e.g. *F*, *t*, *r*) with confidence intervals, effect sizes, degrees of freedom and *P* value noted<br>*Give P values as exact values whenever suitable.*
☐	☒	For Bayesian analysis, information on the choice of priors and Markov chain Monte Carlo settings
☐	☒	For hierarchical and complex designs, identification of the appropriate level for tests and full reporting of outcomes
☐	☒	Estimates of effect sizes (e.g. Cohen's *d*, Pearson's *r*), indicating how they were calculated

Our web collection on statistics for biologists contains articles on many of the points above.

Software and code

Policy information about availability of computer code

Data collection	SRA link -- https://www.ncbi.nlm.nih.gov/bioproject/PRJNA986659
Data analysis	Github repository -- https://github.com/dorograce/CydRegenSeq

For manuscripts utilizing custom algorithms or software that are central to the research but not yet described in published literature, software must be made available to editors and reviewers. We strongly encourage code deposition in a community repository (e.g. GitHub). See the Nature Portfolio guidelines for submitting code & software for further information.

Data

Policy information about availability of data

All manuscripts must include a data availability statement. This statement should provide the following information, where applicable:

- Accession codes, unique identifiers, or web links for publicly available datasets
- A description of any restrictions on data availability
- For clinical datasets or third party data, please ensure that the statement adheres to our policy

RNA-seq data is available in the NCBI sequencing read archive (SRA) located through this link https://www.ncbi.nlm.nih.gov/bioproject/PRJNA986659. Code associated with this project is located on our Github page https://github.com/dorograce/CydRegenSeq

Human research participants

Policy information about studies involving human research participants and Sex and Gender in Research.

Reporting on sex and gender	*Use the terms sex (biological attribute) and gender (shaped by social and cultural circumstances) carefully in order to avoid confusing both terms. Indicate if findings apply to only one sex or gender; describe whether sex and gender were considered in study design whether sex and/or gender was determined based on self-reporting or assigned and methods used. Provide in the source data disaggregated sex and gender data where this information has been collected, and consent has been obtained for sharing of individual-level data; provide overall numbers in this Reporting Summary. Please state if this information has not been collected. Report sex- and gender-based analyses where performed, justify reasons for lack of sex- and gender-based analysis.*
Population characteristics	*Describe the covariate-relevant population characteristics of the human research participants (e.g. age, genotypic information, past and current diagnosis and treatment categories). If you filled out the behavioural & social sciences study design questions and have nothing to add here, write "See above."*
Recruitment	*Describe how participants were recruited. Outline any potential self-selection bias or other biases that may be present and how these are likely to impact results.*
Ethics oversight	*Identify the organization(s) that approved the study protocol.*

Note that full information on the approval of the study protocol must also be provided in the manuscript.

Field-specific reporting

Please select the one below that is the best fit for your research. If you are not sure, read the appropriate sections before making your selection.

☒ Life sciences ☐ Behavioural & social sciences ☐ Ecological, evolutionary & environmental sciences

For a reference copy of the document with all sections, see nature.com/documents/nr-reporting-summary-flat.pdf

Life sciences study design

All studies must disclose on these points even when the disclosure is negative.

Sample size	For RNA-seq, 20-30 animals were pooled for each of the 24 samples. For insitu validation, 10-20 animals were placed in each well
Data exclusions	Technical replicates for replicate 3 in the uncut, 24 and 48 hours after bisection time points were collected, but not utilized in the analysis or uploaded to the SRA.
Replication	For RNA-seq, three biological replicates were included for each time point. For in situ validation, the time course experiment was performed twice, yielding two biological replicates.
Randomization	Biological replicates were generated for RNA-seq and in situ hybridization by pooling animals from separate spawns from different animals.
Blinding	Not relevant to study

Reporting for specific materials, systems and methods

We require information from authors about some types of materials, experimental systems and methods used in many studies. Here, indicate whether each material, system or method listed is relevant to your study. If you are not sure if a list item applies to your research, read the appropriate section before selecting a response.

Materials & experimental systems

n/a	Involved in the study	
☐	☒	Antibodies
☒	☐	Eukaryotic cell lines
☒	☐	Palaeontology and archaeology
☐	☒	Animals and other organisms
☒	☐	Clinical data
☒	☐	Dual use research of concern

Methods

n/a	Involved in the study	
☒	☐	ChIP-seq
☒	☐	Flow cytometry
☒	☐	MRI-based neuroimaging

March 2021

Antibodies

Antibodies used	AP-conjugated anti-digoxigenin(DIG) attached to the RNA probe.
Validation	This antibody recognizes the digoxigenin(DIG) attached to the RNA probe.

Animals and other research organisms

Policy information about studies involving animals; ARRIVE guidelines recommended for reporting animal research, and Sex and Gender in Research

Laboratory animals	Mnemiopsis leidyi
Wild animals	Wild lobate stage animals were caught and transported back to the lab. These animals were then transported to buckets away from the light to induce spawning and placed back in their tank after.
Reporting on sex	Species is hermaphroditic
Field-collected samples	Prior to and following spawning wild animals were fed daily and kept in a pseudo kreisel system with full strength sea water under constant light
Ethics oversight	No ethical approval required for this species

Note that full information on the approval of the study protocol must also be provided in the manuscript.
